# Editorial: Community series in the role of CD1- and MR1-restricted T cells in immunity and disease, volume II

**DOI:** 10.3389/fimmu.2024.1490010

**Published:** 2024-09-16

**Authors:** Kazuya Iwabuchi, Luc Van Kaer

**Affiliations:** ^1^ Department of Immunology, Kitasato University School of Medicine, Sagamihara, Japan; ^2^ Department of Pathology, Microbiology and Immunology, Vanderbilt University School of Medicine, Nashville, TN, United States

**Keywords:** CD1, MR1, natural killer T cells, mucosal-associated invariant T cells, nonpeptide antigens, immunity, immune-mediated disease, unconventional T cells

Cluster of differentiation 1 (CD1) and major histocompatibility complex (MHC)-related 1 (MR1) are MHC class I-related proteins that present non-peptidic antigens to subsets of T lymphocytes with innate-like features (1–6). Since publication of volume I of this Community Series, much progress has been made on the functions and therapeutic potential of CD1- and MR1-restricted T cells. Volume II includes five reviews, seven mini reviews, four original research articles, and one brief research article that provide examples of recent developments in this field.

CD1 has four isoforms, termed CD1a, -b, -c, and -d, that present lipid or glycolipid antigens to T cells (7). The primary research article by Szoke-Kovacs et al. provides new insight into the repertoire of lipids associated with these distinct CD1 isoforms, showing binding with a wide variety of lipids that closely mirror the cellular lipidome. Although all isoforms bind with a common set of lipids, each isoform also contains some unique lipid species, suggesting divergent roles in antigen presentation and immune responses. These findings should inform future studies on the capacity of self-lipids to activate CD1-restricted T cells, with potential therapeutic implications.

Many articles contained in volume II focus on CD1d, which presents glycolipid antigens to natural killer T (NKT) cells, most of which express semi-invariant T cell receptors (TCRs) and are thus called invariant NKT (iNKT) cells (5, 6, 8). The mini review by Hayashizaki et al. discusses the remarkable diversity of glycolipid antigens recognized by iNKT cells. They examine the molecular mechanisms by which the TCRs of iNKT cells can react with such a diverse set of glycolipids, how iNKT cells can generate nuanced immune responses against these antigens, and how the information gleaned from these studies can be employed for developing vaccines to augment cellular and humoral immune responses against microbial pathogens. While iNKT cells share their developmental origins with conventional MHC-restricted T cells, they require unique transcriptional programs that are critical for iNKT cell lineage commitment, acquisition of innate-like features, and differentiation of distinct iNKT effector cell subsets (9). The primary research article by Guo et al. explores the mechanisms by which the epigenetic modifier polycomb repressive complex 2 (PRC2) modulates iNKT cell development, employing mice conditionally deficient for the core subunit of PRC2, termed embryonic ectoderm development (Eed), within the T cell lineage. These animals display various iNKT cell alterations, including reduced numbers, disrupted differentiation, increased cell death, a bias towards interleukin (IL)-4 cytokine production, and alterations in the expression of lineage-specific transcription factors. Remarkably, these animals are exquisitely sensitive to iNKT cell-mediated liver injury induced by acetaminophen, highlighting the critical impact of epigenetic regulators on iNKT cell effector cell differentiation and organ-specific disease. The review by Cui et al. discusses the heterogeneity of iNKT cells within different tissues. They review the intra- and extra-thymic mechanisms that result in the generation of distinct iNKT effector cell subsets and their heterogeneity within distinct tissues, such as the thymus, lung, liver, intestine and adipose tissue. They call attention to a circulating NKT cell subset that depends on IL-15 production by thymic epithelial cells for its development. They further explore the relevance of this heterogeneity in iNKT effector cell subsets to the development of cancer immunotherapies.

A number of articles focus on the role of iNKT cells in disease and the development of iNKT cell-based immunotherapies. The review by Rakjashekar et al. discusses the contribution of iNKT cells to protective immunity against viruses, which do not express enzymes for lipid synthesis but can activate iNKT cells by inducing cytokines and/or agonistic self-lipid antigens. They discuss the role of iNKT cells to protective immunity against important human viral infections and the mechanisms employed by some viruses to downregulate CD1d expression and evade iNKT cell activation. These findings are discussed in the context of prophylactic and therapeutic approaches for viral infections. The review by Kumar et al. focuses on the role of iNKT cells in the development of tissue fibrosis during chronic tissue inflammation, including idiopathic pulmonary fibrosis. They review human and mouse studies implicating iNKT cells in the initiation and progression of inflammatory cascades involving neutrophils, macrophages and fibroblasts that ultimately lead to fibrosis development. They conclude that blocking iNKT cell activation may provide a means to prevent fibrosis development. The mini review by Lee et al. discusses the role of CD1d and iNKT cells to the maintenance of intestinal health and the development of gastrointestinal inflammation and disease. They review studies providing evidence for protective or pathogenic roles of iNKT cells in the development of inflammatory bowel disease, the protective effects of glycolipid-mediated iNKT cell activation on murine colitis, and the contribution of intrinsic signaling by CD1d to these activities. It is concluded that NKT cells are promising targets for designing immune therapies for inflammatory bowel disease. The mini review by Satoh and Iwabuchi examines the contribution of NKT cells to obesity-associated tissue inflammation and its metabolic consequences. This type of research has been associated with divergent results obtained by different research groups, likely due to differences in experimental procedures, animal models, and intestinal microbiota of animal colonies (10). The authors discuss the complex interactions of distinct NKT cell subsets with a diverse set of CD1d-expressing cell types in the adipose tissue, and highlight the potential contribution of intrinsic CD1d signaling to the metabolic alterations observed. The primary research article by Yamasaki et al. explores the adjuvant properties of iNKT cells in licensing dendritic cells for antigen presentation to naïve, conventional MHC-restricted T cells. These investigators introduced prostate cancer antigens, together with mRNA for CD1d, into allogeneic cells, and then loaded these cells with the prototypical iNKT cell antigen α-galactosylceramide (α-GalCer), before injecting them into recipient animals that were then challenged with prostate cancer cells. Not only did they find efficient CD8 T cell priming against the introduced (primary) tumor antigens, but also against secondary antigens, via epitope spreading. These CD8 T cell responses displayed both prophylactic and therapeutic activities against prostate tumor progression. The primary research article by Hoo et al. explores the capacity of α-GalCer to modulate the induction of experimental sepsis in mice. They found that treatment of mice with α-GalCer one week prior to sepsis induction attenuates lethality, in a manner involving iNKT cell polarization towards IL-4 and IL-10 production, and expansion of IL-10-producing B cells. From these findings it is concluded that α-GalCer holds promise for immune prophylaxis of sepsis. The mini review by Takami and Motohashi examines the therapeutic potential of adoptively transferred iNKT cells against cancer. Since CD1d is largely monomorphic, both autologous and allogeneic sources of iNKT cells have been explored. The authors review the clinical trials that have employed such approaches, including iNKT cells modified with chimeric antigen receptors (CARs).

MR1 presents vitamin B metabolites and other small molecules to MR1-restricted T cells, most of which express semi-invariant TCRs and are called mucosal-associated invariant T (MAIT) cells (5, 6, 11–13). The mini review by Ito and Yamasaki discusses the regulation of MAIT cells by host-derived rather than microbial antigens. They focus on recent findings that bile acid-derived metabolites can bind MR1 and activate MAIT cells, which may influence their preferential localization and/or maintenance in the liver. Similar mechanisms might be at play to recruit and/or maintain MAIT cells in other tissue locations where they are prevalent. The mini review by Fukui et al. focuses on the functions of MAIT cells in eye diseases. The authors discuss the protective role of MAIT cells in the development of autoimmune uveitis, in a mechanism that likely involves TCR engagement. Since cognate antigen is able to ameliorate clinical symptoms and visual function, the authors conclude that MAIT cell antigens have therapeutic potential in autoimmune uveitis and possibly other inflammatory eye diseases. Yigit et al. review the potential utility of MAIT cells in cancer immunotherapy. They discuss the complex interactions of MAIT cells with the cancer microenvironment, and how these cells can display both tumor-promoting and -suppressing activities. Nevertheless, these cells hold great promise for the development of cancer immunotherapies, for example by programming them with CARs, with the potential of employing allogeneic MAIT cells as off-the-shelf cell therapies against cancer.

A few of the articles included in this Research Topic cover responses mediated by both CD1d- and MR1-restricted αβ T cells, as well as by another subset of unconventional T cells, γδ T cells, which react with a variety of different ligands, including non-peptidic ones (14, 15). The brief research report by N’guessan et al. explores the potential association of human immunodeficiency virus (HIV)-1 vaccine efficacy with the activation of innate immune cells and unconventional T cells. Using human subjects from the Thai phase III HIV-1 vaccine trial (RV144), these investigators found increased iNKT, MAIT and γδ T cell activation following vaccine administration, suggesting that these cells may contribute to vaccine-induced humoral responses. The review paper by Lv et al. discusses the role of unconventional T cells in the central nervous system. They explore the contribution of iNKT, MAIT and γδ T cells in maintaining brain homeostasis, their roles during the development of acute (e.g., stroke and traumatic brain injury) and chronic (e.g., multiple sclerosis) brain injury, and their involvement in the development of neurodegenerative diseases such Alzheimer’s disease, Parkinson’s disease and amyotrophic lateral sclerosis. The mini review by Liu et al. examines the role of innate-like T cells, including NKT, MAIT and γδ T cells, in the pathogenesis of necrotizing enterocolitis (NEC), a life-threatening gastrointestinal disease associated with bacterial invasion that primarily affects preterm babies. They argue that accumulation of NKT and MAIT cells in preterm babies, possibly mediated by microbial dysbiosis, may contribute to NEC development. Thus, blocking the activation of these cells may provide an immunotherapeutic approach to NEC.

In summary, the articles contained in volume II of this Community Series provide a snapshot of current research on the biology of CD1- and MR1-restricted T cells. We hope this work will spur additional research that will ultimately contribute to the development of new immunotherapies.
